# Spectroscopic, crystallographic, and Hirshfeld surface characterization of nine-membered-ring-containing 9-meth­oxy-3,4,5,6-tetra­hydro-1*H*-benzo[*b*]azonine-2,7-dione and its parent tetra­hydro­car­ba­zole

**DOI:** 10.1107/S2056989023007259

**Published:** 2023-08-23

**Authors:** Maritza J. Flores, Brandon Mai, Joseph M. Tanski

**Affiliations:** a Department of Chemistry, Vassar College, Poughkeepsie, NY 12604, USA; Indian Institute of Science Education and Research Bhopal, India

**Keywords:** crystal structure, tetra­hydro­car­ba­zole, benzo[*b*]azonine, nine-membered ring, hydrogen bond

## Abstract

9-Meth­oxy-3,4,5,6-tetra­hydro-1*H*-benzo[*b*]azonine-2,7-dione, C_13_H_15_NO_3_, (I), and 6-meth­oxy-1,2,3,4-tetra­hydro­car­ba­zole, C_13_H_15_NO, (II), represent the structures of a benzoazonine that contains a nine-membered ring and its parent tetra­hydro­car­ba­zole. The mol­ecules of (I) pack together *via* strong amide N—H⋯O hydrogen bonding and weak C—H⋯O inter­actions, whereas the parent tetra­hydro­car­ba­zole (II) packs with C/N—H⋯π inter­actions, as visualized by Hirshfeld surface characterization.

## Abstract 2

9-Meth­oxy-3,4,5,6-tetra­hydro-1*H*-benzo[*b*]azonine-2,7-dione and 6-meth­oxy-1,2,3,4-tetra­hydro­car­ba­zole represent the structures of a benzoazonine that contains a nine-membered ring and its parent tetra­hydro­car­ba­zole. The mol­ecules of the former pack together *via* strong amide N—H⋯O hydrogen bonding and weak C—H⋯O inter­actions, whereas the parent tetra­hydro­car­ba­zole packs with C/N—H⋯π inter­actions, as visualized by Hirshfeld surface characterization.

## 1. Chemical context

The title com­pound 9-meth­oxy-3,4,5,6-tetra­hydro-1*H*-benzo[*b*]azonine-2,7-dione, (I), was obtained as a by-product during the synthesis of 6-meth­oxy-1,2,3,4-tetra­hydro­car­ba­zole, (II). Compound (II) may be prepared by refluxing *p*-meth­oxy­phenyl­hydrazine hydro­chloride with cyclo­hexa­none in methanol and an anti­mony catalyst (Kumar *et al.*, 2014 ▸) or in ethanol with 2,4,6-tri­chloro-1,3,5-triazine as a catalyst (Siddalingamurthy *et al.*, 2013 ▸). After isolating the tetra­hydro­car­ba­zole, the remaining aqueous methanol was set aside in a refrigerator for several days, from which a batch of light-yellow crystalline material was collected and found by X-ray crystallography, as well as spectroscopy, mass spectrometry and elemental analysis, to be the nine-membered-ring-containing com­pound (I). Benzo[*b*]azoninediones have been shown to be accesible *via* the enzymatic oxidative cleavage of indole carbon–carbon double bonds in the presence of hydrogen peroxide (Takemoto *et al.*, 2004 ▸). 
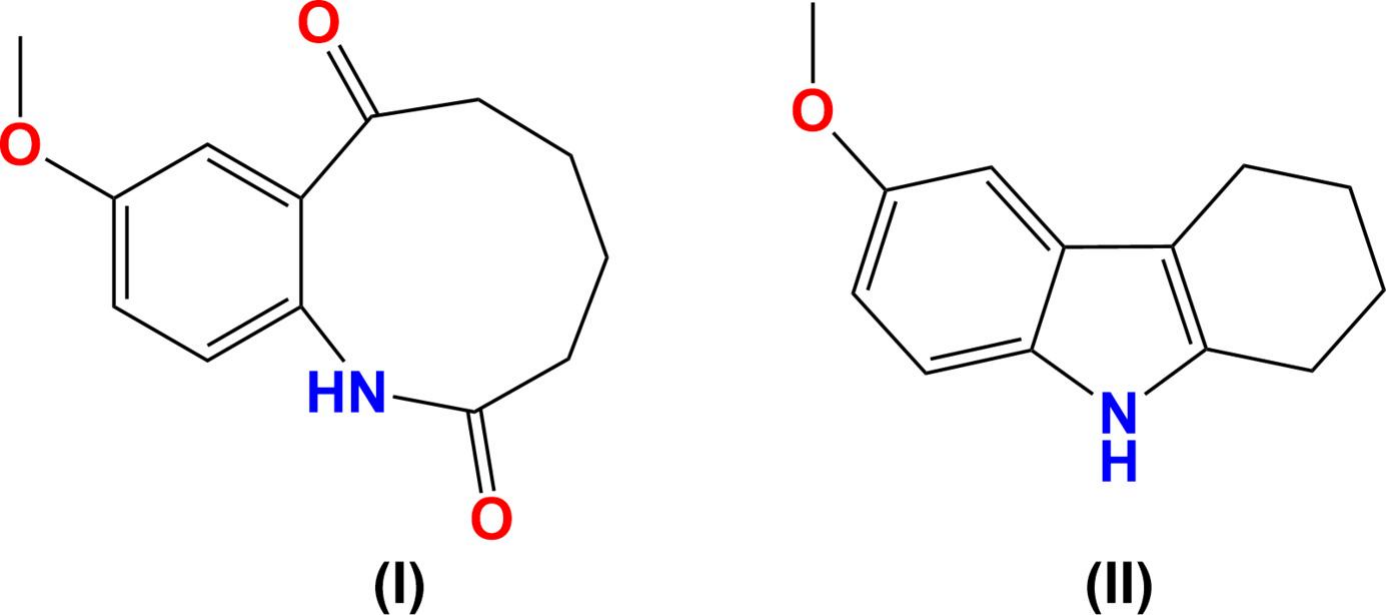


## 2. Structural commentary

**Figure 1:**
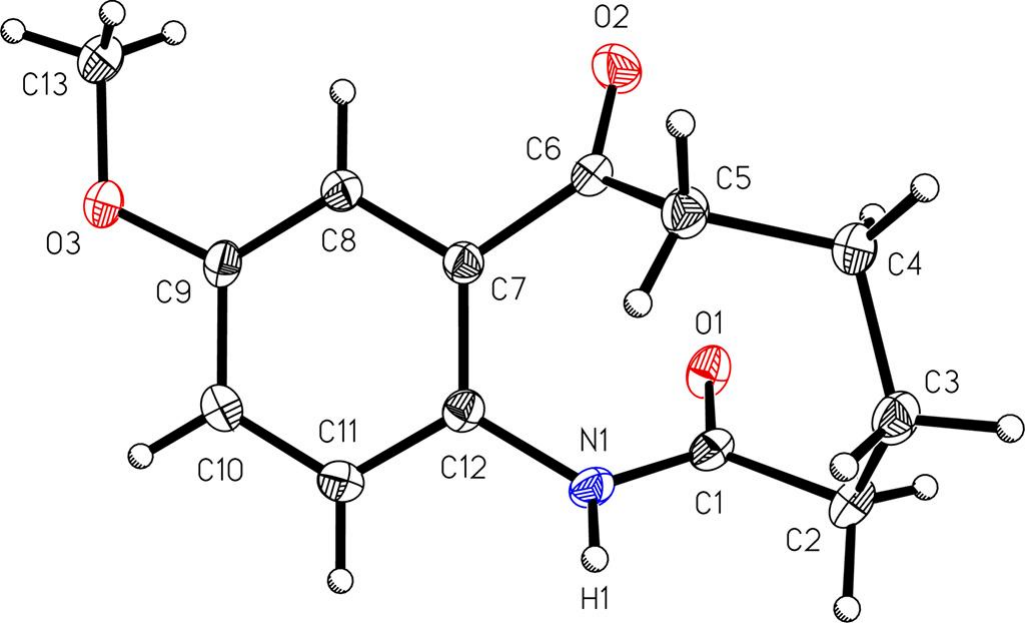
A view of 9-meth­oxy-3,4,5,6-tetra­hydro-1*H*-benzo[*b*]azonine-2,7-dione, (I), with the atom-numbering scheme. Displacement ellipsoids are shown at the 50% probability level.

**Figure 2:**
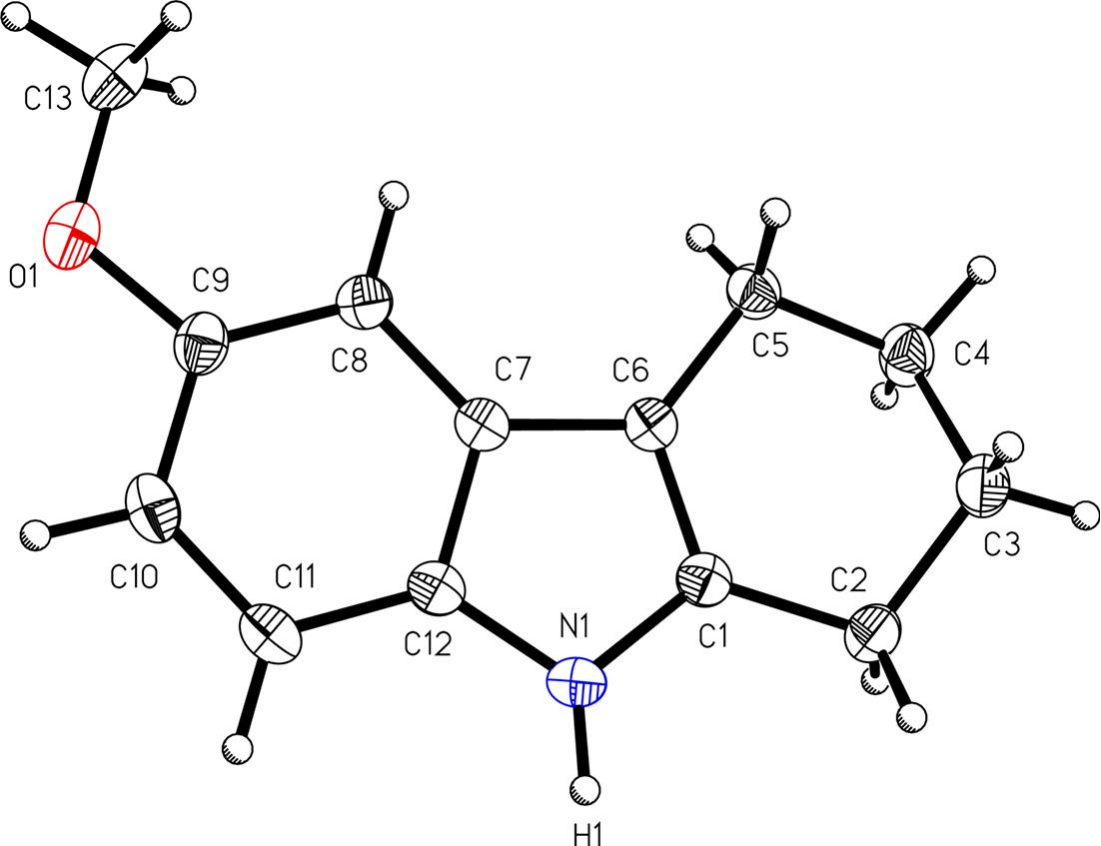
A view of 6-meth­oxy-1,2,3,4-tetra­hydro­car­ba­zole, (II), with the atom-numbering scheme. Displacement ellipsoids are shown at the 50% probability level.

The mol­ecular structure of 9-meth­oxy-3,4,5,6-tetra­hydro-1*H*-benzo[*b*]azonine-2,7-dione, (I) (Fig. 1 ▸), reveals that the mol­ecule contains a nine-membered ring which includes an organic amide and a ketone group. IR spectroscopy corroborates these functional groups with a ketone C=O stretch at 1676 cm^−1^, an amide C=O stretch shifted to lower energy at 1637 cm^−1^, and an amide N—H stretch at 3198 cm^−1^. The structure of the parent com­pound 6-meth­oxy-1,2,3,4-tetra­hydro­car­ba­zole, (II), is shown in Fig. 2 ▸. Unlike related tetra­hydro­car­ba­zoles, such as unsubsituted 1,2,3,4-tetra­hydro­car­ba­zole (McMahon *et al.*, 1997 ▸; Murugavel *et al.*, 2008 ▸; Shukla *et al.*, 2018 ▸), com­pound (II) crystallizes without disorder in the cyclo­hexene ring.

## 3. Supra­molecular features and Hirshfeld surface analysis

**Figure 3:**
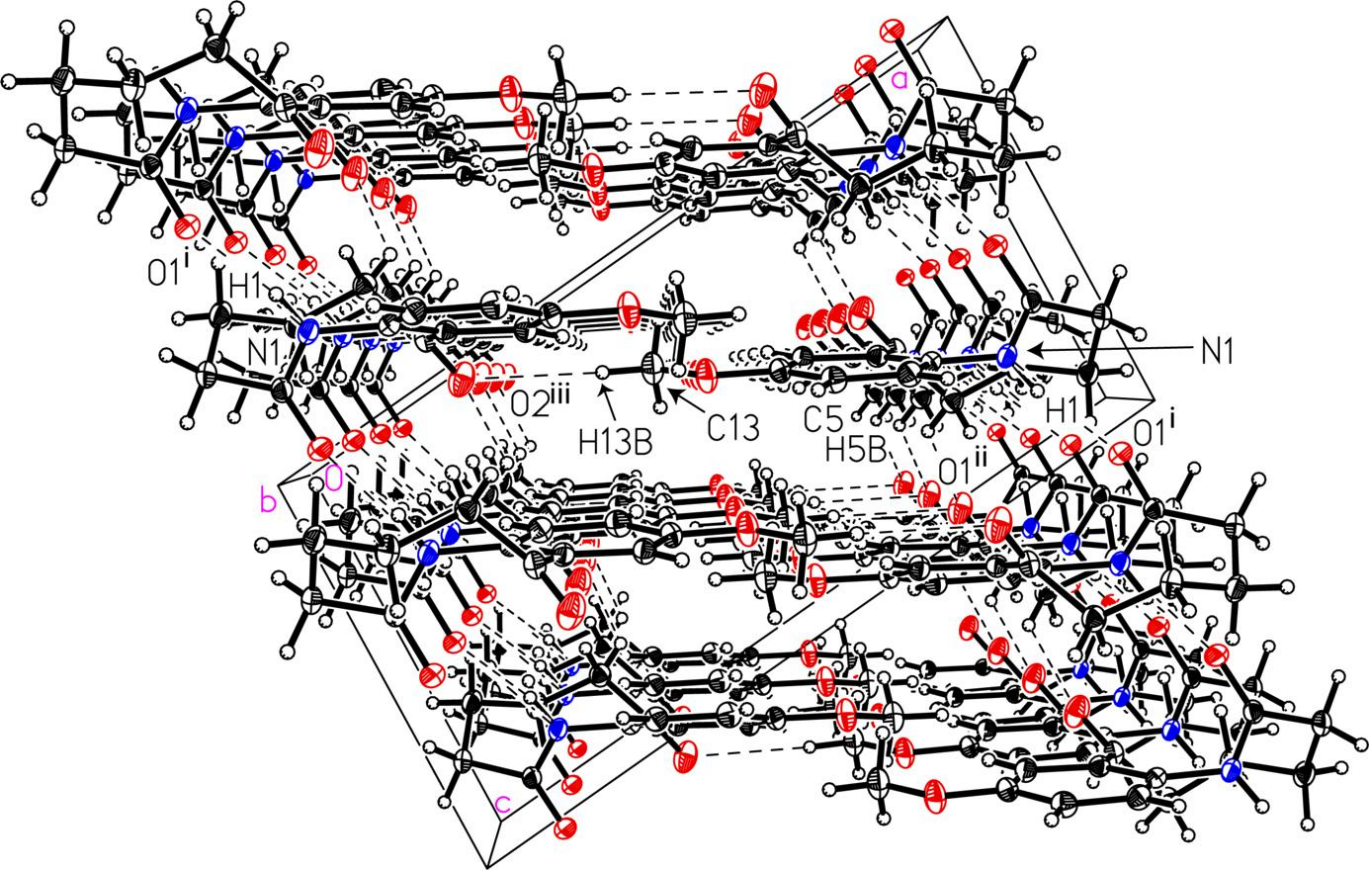
A view of the packing in 9-meth­oxy-3,4,5,6-tetra­hydro-1*H*-benzo[*b*]azonine-2,7-dione, (I). Displacement ellipsoids are shown at the 50% probability level. [Symmetry codes: (i) *x*, −*y* + 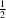 , *z* − 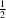 ; (ii) *x*, −*y* + 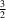 , *z* − 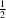 ; (iii) −*x* + 1, *y* − 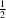 , −*z* + 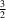 .]

**Figure 4:**
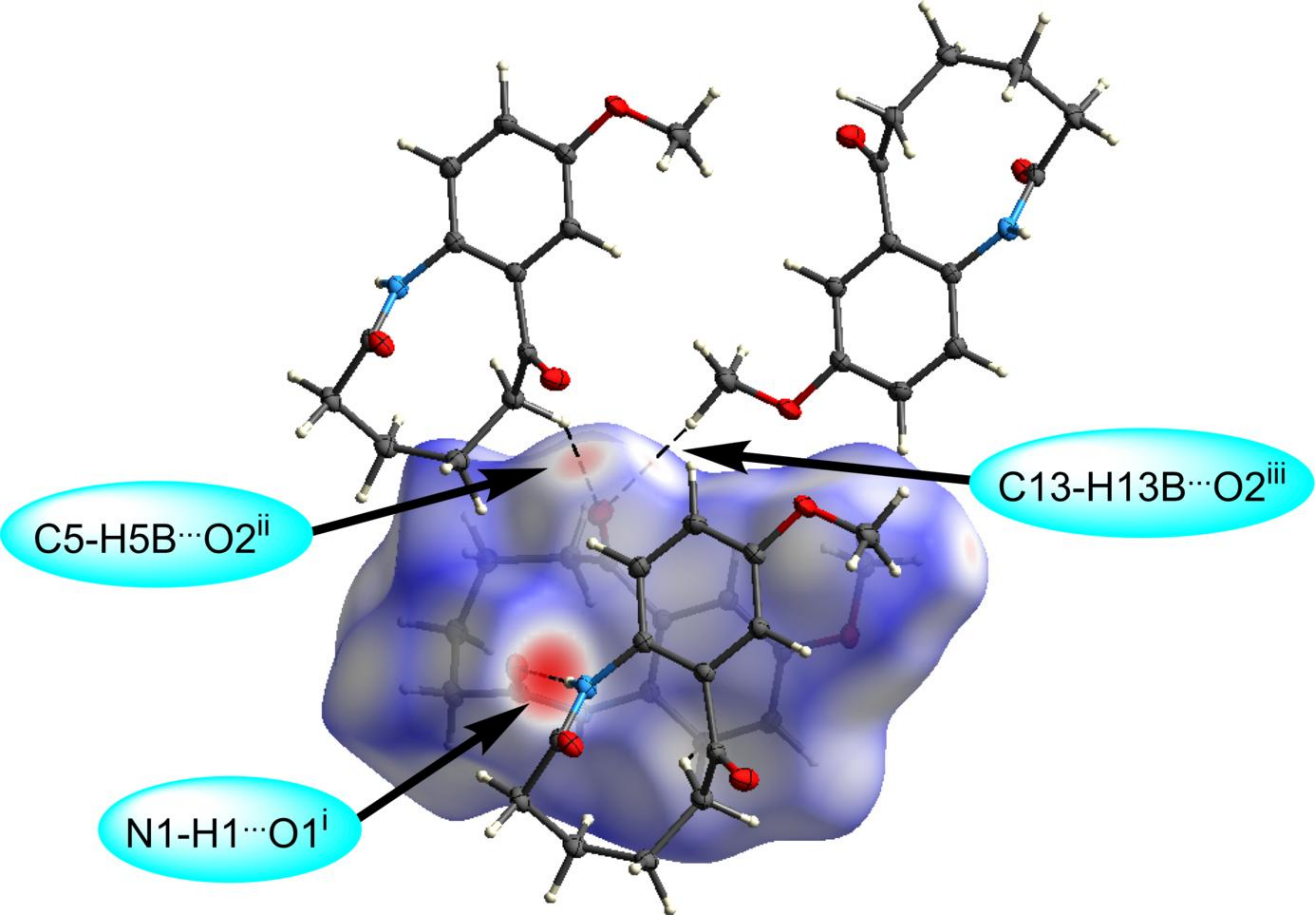
Hirshfeld surface of 9-meth­oxy-3,4,5,6-tetra­hydro-1*H*-benzo[*b*]azonine-2,7-dione, (I), mapped over *d*_norm_, showing the N1—H1⋯O1^i^ hydrogen bond and the weak C5—H5*B*⋯O2^ii^ and C13—H13*B*⋯O2^iii^ inter­actions. [Symmetry codes: (i) *x*, −*y* + 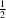 , *z* − 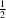 ; (ii) *x*, −*y* + 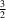 , *z* − 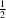 ; (iii) −*x* + 1, *y* − 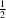 , −*z* + 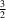 .]

**Figure 5:**
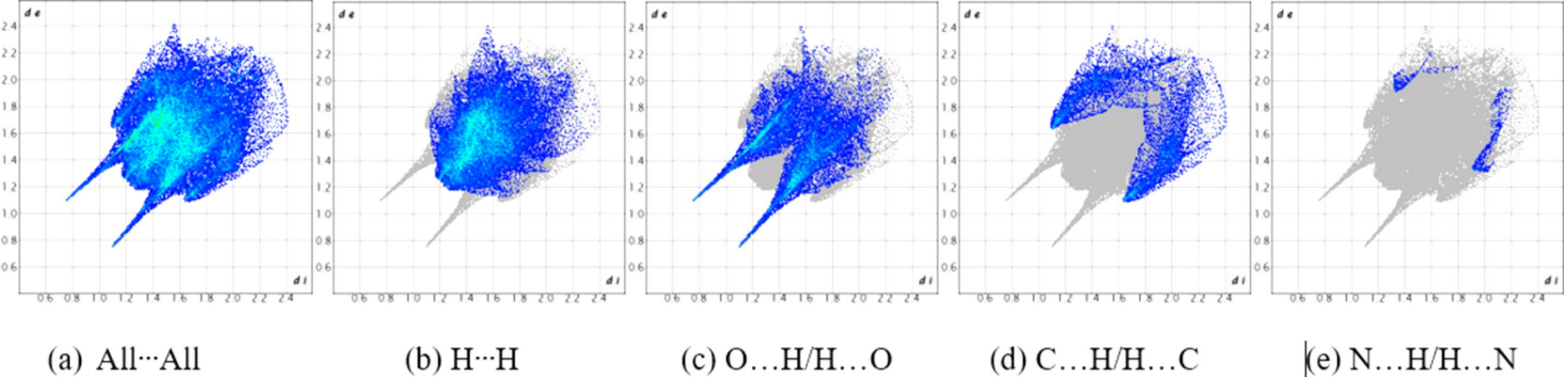
(*a*) The full two-dimensional fingerprint plot for 9-meth­oxy-3,4,5,6-tetra­hydro-1*H*-benzo[*b*]azonine-2,7-dione, (I), and individual fingerprint plots for (*b*) H⋯H (51.3%), (*c*) O⋯H/H⋯O (29.7%), (*d*) C⋯H/H⋯C (15.2%), and (*e*) N⋯H/H⋯N (1.1%) contacts.

The mol­ecules of (I) are held together in the solid state *via* a strong inter­molecular amide N—H⋯O hydrogen bond and weak C—H⋯O inter­actions (Figs. 3 ▸ and 4 ▸, and Table 1 ▸). Specifically, the amide group hydrogen bonds to the O atom of the amide group on a neighboring mol­ecule, *i.e.* N1—H1⋯O1^i^ with a donor–acceptor distance of 2.8426 (12) Å, extending in a one-dimensional chain with graph-set notation *C*(4) (Fig. 3 ▸). The Hirshfeld surface calculated with *CrystalExplorer21* was mapped over *d*_norm_ in the range from −0.5838 to 1.1871 a.u. (Spackman *et al.*, 2021 ▸). The brightest red spot on the surface indicates the N1—H1⋯O1^i^ hydrogen bond, the second most intense spot corresponds to the shorter C5—H5*B*⋯O2^ii^ inter­action, with a hydrogen–acceptor distance of 2.41 Å and a *D*—H⋯*A* angle of 138°, while the least intense spot corresponds to the longer C13—H13*B*⋯O2^iii^ inter­action at a distance of 2.60 Å and with a *D*—H⋯*A* angle of 174° (Fig. 4 ▸ and Table 1 ▸). The two-dimensional fingerprint plots (Fig. 5 ▸) reveal that the most important inter­atomic contacts, summing to 97.3%, are H⋯H (51.3%), O⋯H/H⋯O (29.7%), C⋯H/H⋯C (15.2%), and N⋯H/H⋯N (1.1%). The large percentage contribution and forcep-shaped points in Fig. 5 ▸(*c*) indicate significant O⋯H inter­actions at less than the sum of the van der Waals radii, consistent with the presence of the conventional hydrogen-bond and C—H⋯O inter­actions being abundant points of contact on the surface.

**Figure 6:**
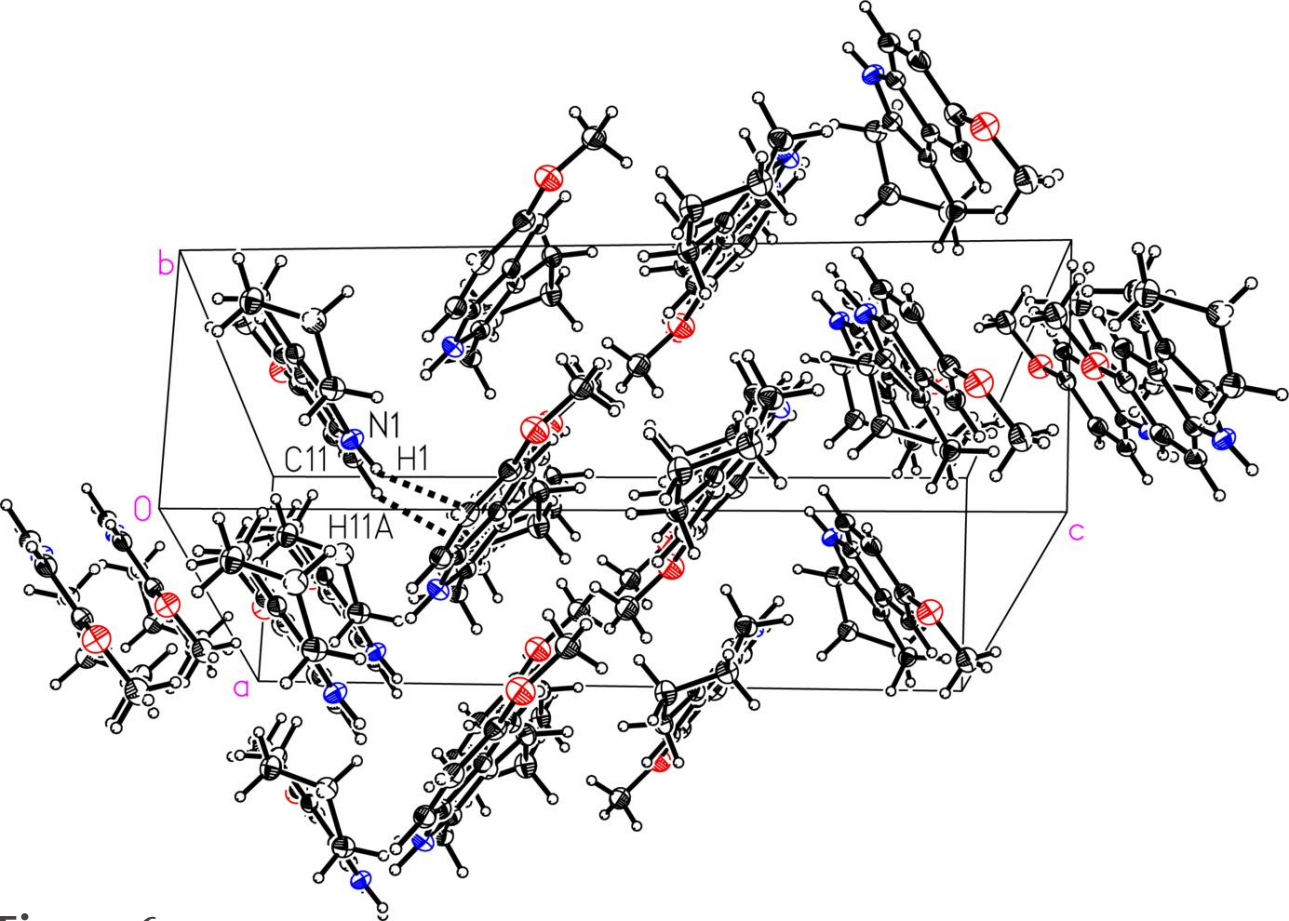
A view of the packing in 6-meth­oxy-1,2,3,4-tetra­hydro­car­ba­zole, (II), showing *via* dashed lines the N1—H1⋯π^i^ and C11—H11*A*⋯π^i^ inter­actions. Displacement ellipsoids are shown at the 50% probability level. [Symmetry code: (i) −*x* + 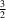 , *y* + 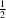 , −*z* + 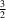 .]

**Figure 7:**
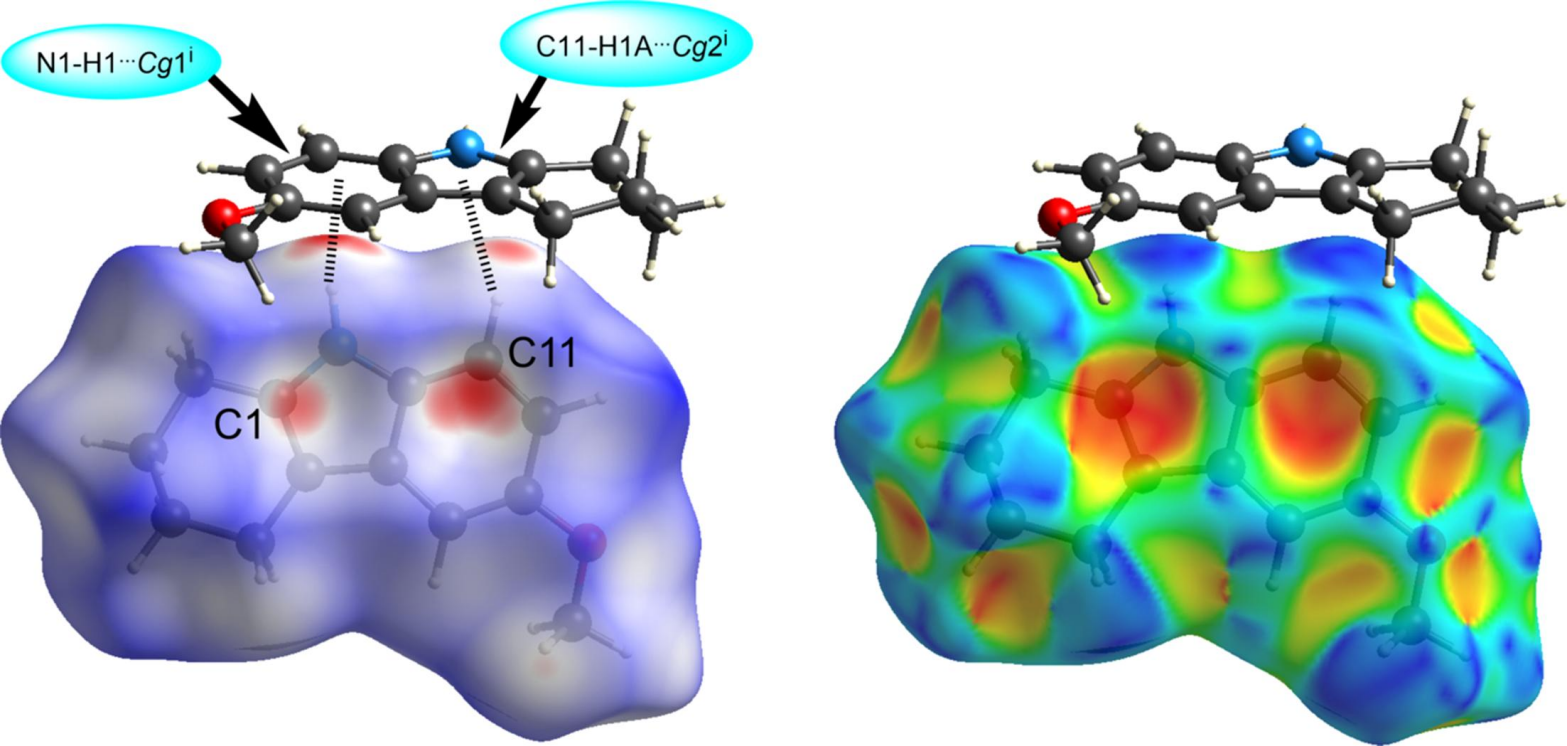
Hirshfeld surface of 6-meth­oxy-1,2,3,4-tetra­hydro­car­ba­zole, (II), mapped over *d*_norm_, showing *via* dashed lines the N1—H1⋯π^i^ and C11—H11*A*⋯π^i^ inter­actions (left), and the surface mapped over the shape-index property. [Symmetry code: (i) −*x* + 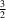 , *y* + 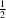 , −*z* + 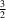 .]

**Figure 8:**
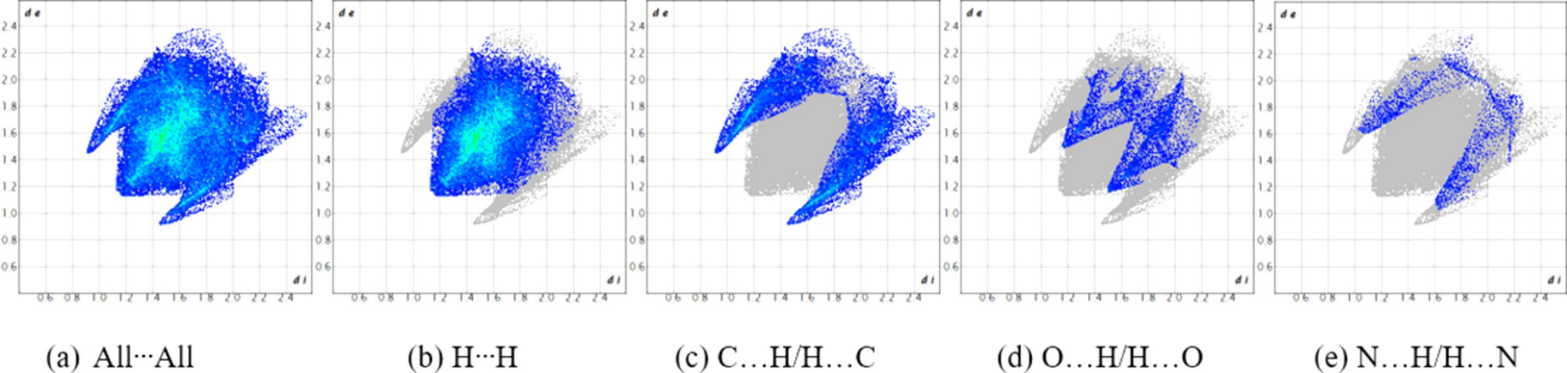
(*a*) The full two-dimensional fingerprint plot for 6-meth­oxy-1,2,3,4-tetra­hydro­car­ba­zole, (II), and individual fingerprint plots for (*b*) H⋯H (63.7%), (*c*) C⋯H/H⋯C (25.5%), (*d*) O⋯H/H⋯O (7.5%), and (*e*) N⋯H/H⋯N (3.3%) contacts.

The mol­ecules of (II) pack with a herringbone motif (Fig. 6 ▸). Although (II) contains an acidic proton, the structure does not exhibit conventional hydrogen bonding, nor any meaningful inter­molecular C—H⋯O/N contacts. However, the Hirshfeld surface calculated with *CrystalExplorer21*, mapped over *d*_norm_ in the range from −0.2999 to 1.3163 a.u. (Spackman *et al.*, 2021 ▸), reveals that the mol­ecules inter­act *via* pairwise N—H⋯π and C—H⋯π inter­actions (Fig. 7 ▸). The brighter red spot on the top left of the surface indicates the N—H⋯π inter­action N1—H1⋯*Cg*1^i^ (Table 2 ▸), which is directed towards the C7–C12 ring on a neighboring mol­ecule, in an offset fashion from the centroid towards C11, with the shortest contact to the ring being C11⋯H1 at a distance of 2.51 Å. The less intense red spot on the top right of the surface indicates the longer C—H⋯π ineraction C11—H11*A*⋯*Cg*2^i^ (Table 2 ▸), which is directed towards the car­ba­zole ring on a neighboring mol­ecule, in an offset fashion from the centroid towards C1, with a C1⋯H11*A* distance of 2.65 Å. The Hirshfeld surface for (II) mapped over the shape-index property further confirms the blue bump shapes of the N/C—H⋯π donors on top and the red valleys of the acceptors on the face (Fig. 7 ▸) (Tan *et al.*, 2019 ▸). The two-dimensional fiingerprint plots (Fig. 8 ▸) show that the most important inter­atomic contacts, summing to 100%, are H⋯H (63.7%), C⋯H/H⋯C (25.5%), O⋯H/H⋯O (7.5%), and N⋯H/H⋯N (3.3%) contacts. The points in the fingerprint plots in Figs. 8 ▸(*b*) and 8(*c*) indicate the significance of H⋯H and C⋯H inter­actions in (II) and the absence of inter­molecular C—H⋯O/N contacts.

## 4. Database survey

A search for com­pounds similar to com­pound (I) in the Cambridge Structural Database (Groom *et al.*, 2016 ▸) found a single structure (CSD refcode COMBEO) which contains the nine-membered ring with an additional acetamide-containing group bridging the 3- and 5-position methyl­ene C atoms of the title com­pound (Baranova *et al.*, 2012 ▸). The additional bridging group in COMBEO positions the amide carbonyl and N—H groups *cis* to one another, with an O—C—N—H torsion angle of 7.37°, allowing for the formation of an 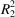 (8) graph-set centrosymmetric hydrogen-bonding dimer, whereas in com­pound (I), they are oriented *trans*, with an O—C—N—H torsion angle of 170.69°, which precludes hydrogen bonding *via* a similar dimer, and (I) forms a one-dimensional hydrogen-bonding chain.

The structure of the unsubsituted 1,2,3,4-tetra­hydro­car­ba­zole has been reported several times [refcodes LOJCIX01 (McMahon *et al.*, 1997 ▸), LOJCIX (Murugavel *et al.*, 2008 ▸), and LOJCIX02 (Shukla *et al.*, 2018 ▸)], together with the simple 1,2,3,4-tetra­hydro­car­ba­zole dervatives substituted at the 6-position with *X* = –F (PIGWOU), –Cl (PIGWAG) or –Br (PIGVIN) (Shukla *et al.*, 2018 ▸), –CO_2_Et (AHEMEF; Hökelek *et al.*, 2002 ▸), and –NHC(O)Ph (MUDWIS; Laitar *et al.*, 2009 ▸). The unsubstituted 1,2,3,4-tetra­hydro­car­ba­zole and its halide derivatives share the same pairwise N—H⋯π and C—H⋯π inter­actions as found in (II), whereas in the –CO_2_Et (AHEMEF) and –NHC(O)Ph (MUDWIS) derivatives, the car­ba­zole N—H group hydrogen bonds inter­molecularly with the carbonyl O atom.

## 5. Synthesis and crystallization

In a fashion similar to that reported previously in the literature (Kumar *et al.*, 2014 ▸), equimolar amounts of (*p*-meth­oxy­phen­yl)hydrazine hydro­chloride (10 mmol, 1.746 g) and cyclo­hexa­none (10 mmol, 1.04 ml) were added to a round-bottomed flask along with 10 mol% anti­mony trioxide as a catalyst (0.001 mol, 0.291 g) in methanol solvent (40 ml). The resulting mixture was refluxed in a mineral oil bath at 338 K overnight. The reaction mixture was then cooled to room temperature and quenched slowly with 10 ml of water and 10 ml of saturated sodium bicarbonate. The aqueous layer was then extracted with ethyl acetate (3 × 30 ml). The combined organic layer was dried overnight with anhydrous MgSO_4_, filtered, and evaporated under reduced pressure, yielding 740 mg (37%) of (II). The ^1^H NMR data matched those reported previously in the literature. After isolating the tetra­hydro­car­ba­zole, the remaining aqueous methanol was set aside in a refrigerator for several days, from which a batch of faint-yellow crystalline material was collected and found by X-ray crystallography, as well as NMR and IR spectroscopy, mass spectrometry, and elemental analysis, to be the nine-membered-ring com­pound 9-meth­oxy-3,4,5,6-tetra­hydro-1*H*-benzo[*b*]azonine-2,7-dione, (I), formed by the oxidative cleavage of the indole carbon–carbon double bond of the parent tetra­hydro­car­ba­zole 6-meth­oxy-1,2,3,4-tetra­hydro­car­ba­zole, (II).

## 6. Refinement

Crystal data, data collection and structure refinement details are summarized in Table 3 ▸. H atoms on C atoms were included in calculated positions and refined using a riding model, with C—H = 0.95 Å and *U*_iso_(H) = 1.2*U*_eq_(C) for aryl H atoms, C—H = 0.98 Å and *U*_iso_(H) = 1.5*U*_eq_(C) for methyl H atoms, and C—H = 0.99 Å and *U*_iso_(H) = 1.2*U*_eq_(C) for methyl­ene H atoms. The positions of the amide H atom in (I) and the amine H atom in (II) were found in difference maps and refined semi-freely using a distance restraint of N—H = 0.88 Å and *U*_iso_(H) = 1.2*U*_eq_(N).

## 7. Analytical data for (I)

^1^H NMR (Bruker Avance III HD 400 MHz, CDCl_3_): δ 1.84 (*m*, 4H, 2 C*H*_2_), 2.25 (*m*, 2H, C*H*_2_), 2.91 (*m*, 2H, C*H*_2_), 3.86 (*s*, 3H, OC*H*_3_), 7.02 (*dd*, 1H, C_ar­yl_ *H*, *J*_ortho_ = 8.6 Hz, *J*_meta_ = 3.0 Hz), 7.05 (*d*, 1H, C_ar­yl_ *H*, *J*_meta_ = 3.0 Hz), 7.16 (*d*, 1H, C_ar­yl_ *H*, *J*_ortho_ = 8.6 Hz), 7.19 (*br s*, 1H, N*H*). ^13^C NMR (^13^C{^1^H}, 100.6 MHz, CDCl_3_): δ 24.42 (*C*H_2_), 25.45 (*C*H_2_), 32.56 (*C*H_2_), 41.83 (*C*H_2_), 55.72 (O*C*H_3_), 112.87 (*C*_ar­yl_H), 117.99 (*C*_ar­yl_H), 126.58 (*C*_ar­yl_), 130.50 (*C*_ar­yl_H), 140.89 (*C*_ar­yl_), 159.37 (*C*_ar­yl_), 176.49 (*C*=O)NH, 205.76 (*C*=O). IR (Thermo Nicolet iS50, ATR, cm^−1^): 3197.85 (*m*, N—H str), 3004.02 (*w*, C_ar­yl_—H str), 2936.23 (*m*, C_alk­yl_—H str), 2860.88 (*w*, C_alk­yl_—H str), 2834.53 (*w*, C_alk­yl_—H str), 1675.96 (*s*, C=O str), 1637.49 (*s*, amide C=O str), 1606.93 (*m*), 1586.93 (*m*), 1519.75 (*m*), 1494.23 (*s*), 1449.73 (*m*), 1436.91 (*s*), 1411.61 (*m*), 1334.69 (*m*), 1274.09 (*s*), 1255.42 (*m*), 1227.85 (*s*), 1208.66 (*s*), 1189.46 (*m*), 1166.34 (*s*), 1139.73 (*s*), 1108.74 (*m*), 1046.53 (*m*), 1031.85 (*s*), 948.63 (*m*), 919.84 (*m*), 895.70 (*m*), 856.17 (*m*), 827.82 (*s*), 811.89 (*m*), 793.28 (*s*), 745.79 (*m*), 718.67 (*m*), 688.02 (*m*), 624.27 (*m*), 604.18 (*m*), 580.24 (*m*), 531.09 (*m*), 497.82 (*s*), 462.99 (*m*), 432.18 (*m*). GC–MS (Agilent Technologies 7890A GC/5975C MS): *M* ^+^ = 233.1 amu. Elemental analysis (CHN) carried out by Robertson Microlit Laboratories, Ledgewood, NJ, USA. Analysis calculated (%) for C_13_H_15_NO_3_: C 66.94, H 6.48, N 6.00; found: C 66.58, H 6.57, N 5.92.

## Supplementary Material

Crystal structure: contains datablock(s) global, I, II. DOI: 10.1107/S2056989023007259/dx2054sup1.cif

Structure factors: contains datablock(s) I. DOI: 10.1107/S2056989023007259/dx2054Isup2.hkl

Structure factors: contains datablock(s) II. DOI: 10.1107/S2056989023007259/dx2054IIsup3.hkl

Supporting information file. DOI: 10.1107/S2056989023007259/dx2054Isup4.cml

Supporting information file. DOI: 10.1107/S2056989023007259/dx2054IIsup5.cml

CCDC references: 2289217, 2289218

Additional supporting information:  crystallographic information; 3D view; checkCIF report

## Figures and Tables

**Table 1:** Hydrogen-bond geometry (Å, °) for (I)

*D*—H⋯*A*	*D*—H	H⋯*A*	*D*⋯*A*	*D*—H⋯*A*
N1—H1⋯O1^i^	0.87 (1)	1.99 (1)	2.8426 (12)	167 (1)
C5—H5*B*⋯O2^ii^	0.99	2.41	3.2085 (14)	138
C13—H13*B*⋯O2^iii^	0.98	2.60	3.5793 (15)	174

Symmetry codes: (i) 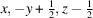 ; (ii) 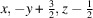 ; (iii) 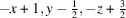 .

**Table 2:** Hydrogen-bond geometry (Å, °) for (II) *Cg*1 and *Cg*2 are the centroids of the C7–C12 and N1/C1/C6/C7/C12 rings, respectively.

*D*—H⋯*A*	*D*—H	H⋯*A*	*D*⋯*A*	*D*—H⋯*A*
N1—H1⋯*Cg*1^i^	0.88 (1)	2.41 (1)	3.2645 (11)	150
C11—H11*A*⋯*Cg*2^i^	0.95	2.61	3.5018 (12)	146

Symmetry code: (i) 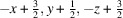 .

**Table 3:** Experimental details Experiments were carried out at 125 K with Mo *K*α radiation using a Bruker APEXII CCD diffractometer. Absorption was corrected for by multi-scan methods (*SADABS*; Bruker, 2013 ▸). Refinement was with 1 restraint. H atoms were treated by a mixture of independent and constrained refinement.

	(I)	(II)
Crystal data		
Chemical formula	C_13_H_15_NO_3_	C_13_H_15_NO
*M*_r_	233.26	201.26
Crystal system, space group	Monoclinic, *P*2_1_/*c*	Monoclinic, *C*2/*c*
*a*, *b*, *c* (Å)	16.0139 (8), 8.2743 (4), 8.5596 (4)	20.513 (2), 5.6374 (6), 18.783 (2)
β (°)	96.484 (1)	100.757 (2)
*V* (Å^3^)	1126.92 (9)	2133.9 (4)
*Z*	4	8
μ (mm^−1^)	0.10	0.08
Crystal size (mm)	0.40 × 0.10 × 0.03	0.21 × 0.10 × 0.10

Data collection		
*T*_min_, *T*_max_	0.91, 1.00	0.93, 0.99
No. of measured, independent and observed [*I* > 2σ(*I*)] reflections	27192, 3432, 2685	24248, 3249, 2619
*R*_int_	0.038	0.030
(sin θ/λ)_max_ (Å^−1^)	0.715	0.715

Refinement		
*R*[*F* ^2^ > 2σ(*F* ^2^)], *wR*(*F* ^2^), *S*	0.040, 0.110, 1.04	0.042, 0.117, 1.03
No. of reflections	3432	3249
No. of parameters	158	140
Δρ_max_, Δρ_min_ (e Å^−3^)	0.37, −0.19	0.42, −0.18

Computer programs: *APEX2* (Bruker, 2013 ▸), *SAINT* (Bruker, 2013 ▸), *SHELXT2018* (Sheldrick, 2015*a* ▸), *SHELXL2017* (Sheldrick, 2015*b* ▸), *SHELXTL2014* (Sheldrick, 2008 ▸), *OLEX2* (Dolomanov *et al.*, 2009 ▸), and *Mercury* (Macrae *et al.*, 2020 ▸).

## supplementary crystallographic information

### 9-Methoxy-3,4,5,6-tetrahydro-1H-benzo[b]azonine-2,7-dione (I) Crystal data

**Table:**

C_13_H_15_NO_3_	*F*(000) = 496
*M**_r_* = 233.26	*D*_x_ = 1.375 Mg m^−^^3^
Monoclinic, *P*2_1_/*c*	Mo *K*α radiation, λ = 0.71073 Å
*a* = 16.0139 (8) Å	Cell parameters from 9966 reflections
*b* = 8.2743 (4) Å	θ = 2.6–30.5°
*c* = 8.5596 (4) Å	µ = 0.10 mm^−^^1^
β = 96.484 (1)°	*T* = 125 K
*V* = 1126.92 (9) Å^3^	Needle, yellow
*Z* = 4	0.40 × 0.10 × 0.03 mm

### 9-Methoxy-3,4,5,6-tetrahydro-1H-benzo[b]azonine-2,7-dione (I) Data collection

**Table:**

Bruker APEXII CCD diffractometer	3432 independent reflections
Radiation source: sealed X-ray tube, Bruker APEXII CCD	2685 reflections with *I* > 2σ(*I*)
Graphite monochromator	*R*_int_ = 0.038
Detector resolution: 8.3333 pixels mm^-1^	θ_max_ = 30.5°, θ_min_ = 2.6°
φ and ω scans	*h* = −22→22
Absorption correction: multi-scan (SADABS; Bruker, 2013)	*k* = −11→11
*T*_min_ = 0.91, *T*_max_ = 1.00	*l* = −12→12
27192 measured reflections	

### 9-Methoxy-3,4,5,6-tetrahydro-1H-benzo[b]azonine-2,7-dione (I) Refinement

**Table:**

Refinement on *F*^2^	Primary atom site location: dual
Least-squares matrix: full	Secondary atom site location: difference Fourier map
*R*[*F*^2^ > 2σ(*F*^2^)] = 0.040	Hydrogen site location: mixed
*wR*(*F*^2^) = 0.110	H atoms treated by a mixture of independent and constrained refinement
*S* = 1.04	*w* = 1/[σ^2^(*F*_o_^2^) + (0.0545*P*)^2^ + 0.3033*P*] where *P* = (*F*_o_^2^ + 2*F*_c_^2^)/3
3432 reflections	(Δ/σ)_max_ < 0.001
158 parameters	Δρ_max_ = 0.37 e Å^−^^3^
1 restraint	Δρ_min_ = −0.19 e Å^−^^3^

### 9-Methoxy-3,4,5,6-tetrahydro-1H-benzo[b]azonine-2,7-dione (I) Special details

**Table:**

Geometry. All esds (except the esd in the dihedral angle between two l.s. planes) are estimated using the full covariance matrix. The cell esds are taken into account individually in the estimation of esds in distances, angles and torsion angles; correlations between esds in cell parameters are only used when they are defined by crystal symmetry. An approximate (isotropic) treatment of cell esds is used for estimating esds involving l.s. planes.

### 9-Methoxy-3,4,5,6-tetrahydro-1H-benzo[b]azonine-2,7-dione (I) Fractional atomic coordinates and isotropic or equivalent isotropic displacement parameters (Å^2^)

**Table:**

	*x*	*y*	*z*	*U*_iso_*/*U*_eq_	
O1	0.11242 (5)	0.38292 (11)	0.51840 (9)	0.02220 (19)	
O2	0.27855 (6)	0.62150 (11)	0.56201 (10)	0.0280 (2)	
O3	0.47875 (5)	0.12590 (10)	0.65022 (10)	0.0253 (2)	
N1	0.16638 (6)	0.25619 (12)	0.31640 (10)	0.01783 (19)	
H1	0.1564 (9)	0.2229 (17)	0.2199 (14)	0.021*	
C1	0.10773 (7)	0.34800 (14)	0.37727 (12)	0.0177 (2)	
C2	0.04082 (7)	0.41965 (15)	0.26000 (13)	0.0213 (2)	
H2A	−0.005196	0.463892	0.31533	0.026*	
H2B	0.017052	0.33484	0.186381	0.026*	
C3	0.07963 (7)	0.55581 (15)	0.16766 (13)	0.0217 (2)	
H3A	0.106874	0.50615	0.081181	0.026*	
H3B	0.033617	0.625756	0.119347	0.026*	
C4	0.14461 (7)	0.66276 (14)	0.26519 (13)	0.0218 (2)	
H4A	0.125394	0.681824	0.369587	0.026*	
H4B	0.147046	0.768829	0.21239	0.026*	
C5	0.23445 (7)	0.58971 (14)	0.28901 (13)	0.0204 (2)	
H5A	0.236483	0.499036	0.214073	0.024*	
H5B	0.274181	0.67316	0.259799	0.024*	
C6	0.26634 (7)	0.52791 (14)	0.45201 (13)	0.0181 (2)	
C7	0.29362 (7)	0.35427 (13)	0.47129 (12)	0.0163 (2)	
C8	0.37105 (7)	0.32367 (13)	0.55989 (12)	0.0181 (2)	
H8	0.401666	0.409546	0.613125	0.022*	
C9	0.40292 (7)	0.16727 (14)	0.56963 (12)	0.0186 (2)	
C10	0.35732 (7)	0.04099 (14)	0.49279 (13)	0.0208 (2)	
H10	0.379971	−0.065242	0.496916	0.025*	
C11	0.27931 (7)	0.06986 (14)	0.41066 (13)	0.0196 (2)	
H11	0.247791	−0.017201	0.361538	0.024*	
C12	0.24680 (6)	0.22611 (13)	0.39972 (12)	0.0161 (2)	
C13	0.52750 (8)	0.25286 (16)	0.72746 (15)	0.0268 (3)	
H13A	0.540945	0.332863	0.649661	0.04*	
H13B	0.579679	0.20819	0.78132	0.04*	
H13C	0.495377	0.304782	0.804483	0.04*	

### 9-Methoxy-3,4,5,6-tetrahydro-1H-benzo[b]azonine-2,7-dione (I) Atomic displacement parameters (Å^2^)

**Table:**

	*U*^11^	*U*^22^	*U*^33^	*U*^12^	*U*^13^	*U*^23^
O1	0.0201 (4)	0.0315 (5)	0.0149 (3)	0.0040 (3)	0.0016 (3)	0.0014 (3)
O2	0.0317 (5)	0.0223 (4)	0.0274 (4)	0.0051 (3)	−0.0073 (4)	−0.0077 (3)
O3	0.0179 (4)	0.0227 (4)	0.0332 (5)	0.0053 (3)	−0.0061 (3)	−0.0022 (3)
N1	0.0171 (4)	0.0211 (5)	0.0143 (4)	−0.0007 (3)	−0.0023 (3)	−0.0025 (3)
C1	0.0147 (5)	0.0205 (5)	0.0174 (5)	−0.0031 (4)	0.0000 (4)	0.0022 (4)
C2	0.0155 (5)	0.0287 (6)	0.0188 (5)	0.0002 (4)	−0.0027 (4)	0.0017 (4)
C3	0.0209 (5)	0.0249 (6)	0.0179 (5)	0.0018 (4)	−0.0032 (4)	0.0026 (4)
C4	0.0209 (5)	0.0205 (5)	0.0228 (5)	0.0027 (4)	−0.0036 (4)	0.0018 (4)
C5	0.0194 (5)	0.0203 (5)	0.0212 (5)	0.0008 (4)	0.0006 (4)	0.0032 (4)
C6	0.0140 (5)	0.0186 (5)	0.0212 (5)	0.0002 (4)	−0.0008 (4)	−0.0012 (4)
C7	0.0162 (5)	0.0171 (5)	0.0156 (4)	0.0013 (4)	0.0013 (4)	−0.0011 (4)
C8	0.0167 (5)	0.0184 (5)	0.0187 (5)	0.0009 (4)	0.0001 (4)	−0.0023 (4)
C9	0.0149 (5)	0.0214 (5)	0.0193 (5)	0.0033 (4)	0.0007 (4)	0.0004 (4)
C10	0.0211 (5)	0.0177 (5)	0.0237 (5)	0.0032 (4)	0.0028 (4)	−0.0011 (4)
C11	0.0207 (5)	0.0178 (5)	0.0202 (5)	−0.0010 (4)	0.0017 (4)	−0.0029 (4)
C12	0.0157 (5)	0.0194 (5)	0.0132 (4)	0.0005 (4)	0.0013 (3)	−0.0005 (4)
C13	0.0196 (5)	0.0281 (6)	0.0309 (6)	0.0020 (5)	−0.0053 (5)	−0.0029 (5)

### 9-Methoxy-3,4,5,6-tetrahydro-1H-benzo[b]azonine-2,7-dione (I) Geometric parameters (Å, º)

**Table:**

O1—C1	1.2361 (13)	C5—C6	1.5192 (15)
O2—C6	1.2177 (13)	C5—H5A	0.99
O3—C9	1.3705 (13)	C5—H5B	0.99
O3—C13	1.4260 (15)	C6—C7	1.5053 (15)
N1—C1	1.3570 (14)	C7—C12	1.3995 (15)
N1—C12	1.4218 (14)	C7—C8	1.4015 (15)
N1—H1	0.869 (12)	C8—C9	1.3901 (16)
C1—C2	1.5047 (15)	C8—H8	0.95
C2—C3	1.5463 (17)	C9—C10	1.3961 (16)
C2—H2A	0.99	C10—C11	1.3834 (16)
C2—H2B	0.99	C10—H10	0.95
C3—C4	1.5384 (16)	C11—C12	1.3930 (15)
C3—H3A	0.99	C11—H11	0.95
C3—H3B	0.99	C13—H13A	0.98
C4—C5	1.5526 (16)	C13—H13B	0.98
C4—H4A	0.99	C13—H13C	0.98
C4—H4B	0.99		
			
C9—O3—C13	117.19 (9)	C4—C5—H5B	107.9
C1—N1—C12	122.17 (9)	H5A—C5—H5B	107.2
C1—N1—H1	118.8 (9)	O2—C6—C7	120.19 (10)
C12—N1—H1	118.8 (9)	O2—C6—C5	120.27 (10)
O1—C1—N1	122.53 (10)	C7—C6—C5	119.03 (9)
O1—C1—C2	121.33 (10)	C12—C7—C8	119.84 (10)
N1—C1—C2	115.85 (9)	C12—C7—C6	122.73 (9)
C1—C2—C3	109.33 (9)	C8—C7—C6	117.38 (9)
C1—C2—H2A	109.8	C9—C8—C7	119.81 (10)
C3—C2—H2A	109.8	C9—C8—H8	120.1
C1—C2—H2B	109.8	C7—C8—H8	120.1
C3—C2—H2B	109.8	O3—C9—C8	124.13 (10)
H2A—C2—H2B	108.3	O3—C9—C10	115.90 (10)
C4—C3—C2	115.34 (9)	C8—C9—C10	119.97 (10)
C4—C3—H3A	108.4	C11—C10—C9	120.30 (10)
C2—C3—H3A	108.4	C11—C10—H10	119.9
C4—C3—H3B	108.4	C9—C10—H10	119.9
C2—C3—H3B	108.4	C10—C11—C12	120.24 (10)
H3A—C3—H3B	107.5	C10—C11—H11	119.9
C3—C4—C5	114.03 (10)	C12—C11—H11	119.9
C3—C4—H4A	108.7	C11—C12—C7	119.74 (10)
C5—C4—H4A	108.7	C11—C12—N1	120.44 (10)
C3—C4—H4B	108.7	C7—C12—N1	119.81 (10)
C5—C4—H4B	108.7	O3—C13—H13A	109.5
H4A—C4—H4B	107.6	O3—C13—H13B	109.5
C6—C5—C4	117.52 (9)	H13A—C13—H13B	109.5
C6—C5—H5A	107.9	O3—C13—H13C	109.5
C4—C5—H5A	107.9	H13A—C13—H13C	109.5
C6—C5—H5B	107.9	H13B—C13—H13C	109.5
			
C12—N1—C1—O1	−15.63 (16)	C13—O3—C9—C8	−0.42 (16)
C12—N1—C1—C2	158.31 (10)	C13—O3—C9—C10	178.85 (10)
O1—C1—C2—C3	102.43 (12)	C7—C8—C9—O3	178.48 (10)
N1—C1—C2—C3	−71.59 (13)	C7—C8—C9—C10	−0.76 (16)
C1—C2—C3—C4	−38.86 (13)	O3—C9—C10—C11	178.79 (10)
C2—C3—C4—C5	82.68 (12)	C8—C9—C10—C11	−1.91 (16)
C3—C4—C5—C6	−108.22 (11)	C9—C10—C11—C12	2.07 (17)
C4—C5—C6—O2	−65.85 (14)	C10—C11—C12—C7	0.44 (16)
C4—C5—C6—C7	122.35 (11)	C10—C11—C12—N1	−179.54 (10)
O2—C6—C7—C12	142.88 (11)	C8—C7—C12—C11	−3.09 (15)
C5—C6—C7—C12	−45.31 (14)	C6—C7—C12—C11	174.17 (10)
O2—C6—C7—C8	−39.79 (15)	C8—C7—C12—N1	176.89 (9)
C5—C6—C7—C8	132.02 (10)	C6—C7—C12—N1	−5.85 (15)
C12—C7—C8—C9	3.25 (16)	C1—N1—C12—C11	132.57 (11)
C6—C7—C8—C9	−174.16 (10)	C1—N1—C12—C7	−47.40 (14)

### 9-Methoxy-3,4,5,6-tetrahydro-1H-benzo[b]azonine-2,7-dione (I) Hydrogen-bond geometry (Å, º)

**Table:**

*D*—H···*A*	*D*—H	H···*A*	*D*···*A*	*D*—H···*A*
N1—H1···O1^i^	0.87 (1)	1.99 (1)	2.8426 (12)	167 (1)
C5—H5*B*···O2^ii^	0.99	2.41	3.2085 (14)	138
C13—H13*B*···O2^iii^	0.98	2.60	3.5793 (15)	174

Symmetry codes: (i) *x*, −*y*+1/2, *z*−1/2; (ii) *x*, −*y*+3/2, *z*−1/2; (iii) −*x*+1, *y*−1/2, −*z*+3/2.

### 6-Methoxy-1,2,3,4-tetrahydrocarbazole (II) Crystal data

**Table:**

C_13_H_15_NO	*F*(000) = 864
*M**_r_* = 201.26	*D*_x_ = 1.253 Mg m^−^^3^
Monoclinic, *C*2/*c*	Mo *K*α radiation, λ = 0.71073 Å
*a* = 20.513 (2) Å	Cell parameters from 7271 reflections
*b* = 5.6374 (6) Å	θ = 2.2–30.5°
*c* = 18.783 (2) Å	µ = 0.08 mm^−^^1^
β = 100.757 (2)°	*T* = 125 K
*V* = 2133.9 (4) Å^3^	Block, yellow
*Z* = 8	0.21 × 0.10 × 0.10 mm

### 6-Methoxy-1,2,3,4-tetrahydrocarbazole (II) Data collection

**Table:**

Bruker APEXII CCD diffractometer	3249 independent reflections
Radiation source: sealed X-ray tube, Bruker APEXII CCD	2619 reflections with *I* > 2σ(*I*)
Graphite monochromator	*R*_int_ = 0.030
Detector resolution: 8.3333 pixels mm^-1^	θ_max_ = 30.5°, θ_min_ = 2.0°
φ and ω scans	*h* = −29→29
Absorption correction: multi-scan (SADABS; Bruker, 2013)	*k* = −8→8
*T*_min_ = 0.93, *T*_max_ = 0.99	*l* = −26→26
24248 measured reflections	

### 6-Methoxy-1,2,3,4-tetrahydrocarbazole (II) Refinement

**Table:**

Refinement on *F*^2^	Primary atom site location: dual
Least-squares matrix: full	Secondary atom site location: difference Fourier map
*R*[*F*^2^ > 2σ(*F*^2^)] = 0.042	Hydrogen site location: mixed
*wR*(*F*^2^) = 0.117	H atoms treated by a mixture of independent and constrained refinement
*S* = 1.03	*w* = 1/[σ^2^(*F*_o_^2^) + (0.0616*P*)^2^ + 1.016*P*] where *P* = (*F*_o_^2^ + 2*F*_c_^2^)/3
3249 reflections	(Δ/σ)_max_ = 0.001
140 parameters	Δρ_max_ = 0.42 e Å^−^^3^
1 restraint	Δρ_min_ = −0.18 e Å^−^^3^

### 6-Methoxy-1,2,3,4-tetrahydrocarbazole (II) Special details

**Table:**

Geometry. All esds (except the esd in the dihedral angle between two l.s. planes) are estimated using the full covariance matrix. The cell esds are taken into account individually in the estimation of esds in distances, angles and torsion angles; correlations between esds in cell parameters are only used when they are defined by crystal symmetry. An approximate (isotropic) treatment of cell esds is used for estimating esds involving l.s. planes.

### 6-Methoxy-1,2,3,4-tetrahydrocarbazole (II) Fractional atomic coordinates and isotropic or equivalent isotropic displacement parameters (Å^2^)

**Table:**

	*x*	*y*	*z*	*U*_iso_*/*U*_eq_	
O1	0.87890 (4)	0.58856 (15)	0.58257 (5)	0.0314 (2)	
N1	0.68765 (4)	1.06336 (16)	0.69736 (5)	0.02345 (19)	
H1	0.6884 (6)	1.180 (2)	0.7285 (7)	0.028*	
C1	0.63171 (5)	0.93481 (17)	0.66796 (5)	0.0209 (2)	
C2	0.56378 (5)	0.97953 (19)	0.68306 (6)	0.0262 (2)	
H2A	0.543971	1.11983	0.655501	0.031*	
H2B	0.566248	1.011127	0.735341	0.031*	
C3	0.52072 (5)	0.7590 (2)	0.66027 (6)	0.0269 (2)	
H3A	0.533725	0.631075	0.696225	0.032*	
H3B	0.473584	0.798258	0.659681	0.032*	
C4	0.52847 (5)	0.6710 (2)	0.58520 (6)	0.0275 (2)	
H4A	0.516861	0.800874	0.549615	0.033*	
H4B	0.497181	0.538507	0.570449	0.033*	
C5	0.59931 (5)	0.58670 (19)	0.58405 (6)	0.0232 (2)	
H5A	0.606042	0.426696	0.605908	0.028*	
H5B	0.606091	0.57628	0.533321	0.028*	
C6	0.64860 (5)	0.75580 (17)	0.62552 (5)	0.01940 (19)	
C7	0.71886 (5)	0.76989 (17)	0.62904 (5)	0.01899 (19)	
C8	0.76363 (5)	0.63638 (18)	0.59683 (5)	0.0215 (2)	
H8A	0.74878	0.505574	0.566186	0.026*	
C9	0.83002 (5)	0.70122 (18)	0.61111 (5)	0.0229 (2)	
C10	0.85231 (5)	0.89428 (19)	0.65691 (6)	0.0246 (2)	
H10A	0.898194	0.93291	0.666406	0.03*	
C11	0.80883 (5)	1.02892 (18)	0.68837 (6)	0.0241 (2)	
H11A	0.824024	1.160428	0.718572	0.029*	
C12	0.74189 (5)	0.96489 (17)	0.67422 (5)	0.02058 (19)	
C13	0.85810 (6)	0.4022 (2)	0.53217 (6)	0.0298 (2)	
H13A	0.896092	0.346117	0.512031	0.045*	
H13B	0.823643	0.461442	0.492906	0.045*	
H13C	0.840275	0.271001	0.556907	0.045*	

### 6-Methoxy-1,2,3,4-tetrahydrocarbazole (II) Atomic displacement parameters (Å^2^)

**Table:**

	*U*^11^	*U*^22^	*U*^33^	*U*^12^	*U*^13^	*U*^23^
O1	0.0224 (4)	0.0352 (4)	0.0378 (4)	0.0034 (3)	0.0089 (3)	−0.0030 (3)
N1	0.0257 (4)	0.0205 (4)	0.0244 (4)	−0.0018 (3)	0.0053 (3)	−0.0051 (3)
C1	0.0224 (5)	0.0199 (4)	0.0205 (4)	−0.0002 (3)	0.0037 (3)	0.0013 (3)
C2	0.0260 (5)	0.0254 (5)	0.0291 (5)	0.0012 (4)	0.0099 (4)	−0.0014 (4)
C3	0.0232 (5)	0.0307 (5)	0.0280 (5)	−0.0024 (4)	0.0080 (4)	−0.0010 (4)
C4	0.0211 (5)	0.0348 (6)	0.0259 (5)	−0.0015 (4)	0.0025 (4)	−0.0021 (4)
C5	0.0212 (4)	0.0255 (5)	0.0223 (5)	−0.0025 (4)	0.0024 (4)	−0.0027 (4)
C6	0.0199 (4)	0.0204 (4)	0.0174 (4)	−0.0001 (3)	0.0021 (3)	0.0011 (3)
C7	0.0208 (4)	0.0187 (4)	0.0168 (4)	−0.0006 (3)	0.0018 (3)	0.0016 (3)
C8	0.0220 (5)	0.0212 (4)	0.0207 (4)	0.0008 (4)	0.0028 (3)	−0.0002 (3)
C9	0.0207 (5)	0.0248 (5)	0.0235 (5)	0.0022 (4)	0.0049 (4)	0.0042 (4)
C10	0.0201 (4)	0.0267 (5)	0.0260 (5)	−0.0040 (4)	0.0018 (4)	0.0059 (4)
C11	0.0256 (5)	0.0220 (5)	0.0233 (5)	−0.0052 (4)	0.0010 (4)	0.0009 (4)
C12	0.0227 (4)	0.0191 (4)	0.0194 (4)	−0.0010 (3)	0.0025 (3)	0.0013 (3)
C13	0.0307 (5)	0.0322 (6)	0.0282 (5)	0.0082 (4)	0.0097 (4)	0.0025 (4)

### 6-Methoxy-1,2,3,4-tetrahydrocarbazole (II) Geometric parameters (Å, º)

**Table:**

O1—C9	1.3772 (12)	C5—C6	1.4979 (14)
O1—C13	1.4251 (15)	C5—H5A	0.99
N1—C1	1.3822 (13)	C5—H5B	0.99
N1—C12	1.3841 (13)	C6—C7	1.4327 (13)
N1—H1	0.878 (12)	C7—C8	1.4083 (13)
C1—C6	1.3700 (14)	C7—C12	1.4150 (13)
C1—C2	1.4944 (14)	C8—C9	1.3870 (14)
C2—C3	1.5387 (15)	C8—H8A	0.95
C2—H2A	0.99	C9—C10	1.4096 (15)
C2—H2B	0.99	C10—C11	1.3848 (15)
C3—C4	1.5309 (15)	C10—H10A	0.95
C3—H3A	0.99	C11—C12	1.3964 (14)
C3—H3B	0.99	C11—H11A	0.95
C4—C5	1.5329 (15)	C13—H13A	0.98
C4—H4A	0.99	C13—H13B	0.98
C4—H4B	0.99	C13—H13C	0.98
			
C9—O1—C13	116.68 (8)	C4—C5—H5B	109.6
C1—N1—C12	108.69 (8)	H5A—C5—H5B	108.1
C1—N1—H1	124.7 (9)	C1—C6—C7	107.08 (8)
C12—N1—H1	126.4 (9)	C1—C6—C5	123.50 (9)
C6—C1—N1	109.72 (9)	C7—C6—C5	129.41 (9)
C6—C1—C2	125.50 (9)	C8—C7—C12	120.09 (9)
N1—C1—C2	124.73 (9)	C8—C7—C6	132.97 (9)
C1—C2—C3	108.54 (9)	C12—C7—C6	106.93 (8)
C1—C2—H2A	110.0	C9—C8—C7	118.14 (9)
C3—C2—H2A	110.0	C9—C8—H8A	120.9
C1—C2—H2B	110.0	C7—C8—H8A	120.9
C3—C2—H2B	110.0	O1—C9—C8	124.27 (10)
H2A—C2—H2B	108.4	O1—C9—C10	114.63 (9)
C4—C3—C2	111.42 (9)	C8—C9—C10	121.09 (9)
C4—C3—H3A	109.3	C11—C10—C9	121.49 (9)
C2—C3—H3A	109.3	C11—C10—H10A	119.3
C4—C3—H3B	109.3	C9—C10—H10A	119.3
C2—C3—H3B	109.3	C10—C11—C12	117.76 (10)
H3A—C3—H3B	108.0	C10—C11—H11A	121.1
C3—C4—C5	112.03 (9)	C12—C11—H11A	121.1
C3—C4—H4A	109.2	N1—C12—C11	131.00 (10)
C5—C4—H4A	109.2	N1—C12—C7	107.57 (9)
C3—C4—H4B	109.2	C11—C12—C7	121.42 (9)
C5—C4—H4B	109.2	O1—C13—H13A	109.5
H4A—C4—H4B	107.9	O1—C13—H13B	109.5
C6—C5—C4	110.18 (9)	H13A—C13—H13B	109.5
C6—C5—H5A	109.6	O1—C13—H13C	109.5
C4—C5—H5A	109.6	H13A—C13—H13C	109.5
C6—C5—H5B	109.6	H13B—C13—H13C	109.5
			
C12—N1—C1—C6	−0.59 (11)	C12—C7—C8—C9	0.33 (14)
C12—N1—C1—C2	177.03 (9)	C6—C7—C8—C9	178.98 (10)
C6—C1—C2—C3	14.48 (14)	C13—O1—C9—C8	3.57 (15)
N1—C1—C2—C3	−162.77 (10)	C13—O1—C9—C10	−175.98 (9)
C1—C2—C3—C4	−46.60 (12)	C7—C8—C9—O1	−179.13 (9)
C2—C3—C4—C5	63.94 (12)	C7—C8—C9—C10	0.40 (15)
C3—C4—C5—C6	−42.77 (12)	O1—C9—C10—C11	178.46 (9)
N1—C1—C6—C7	0.80 (11)	C8—C9—C10—C11	−1.11 (16)
C2—C1—C6—C7	−176.80 (9)	C9—C10—C11—C12	1.03 (15)
N1—C1—C6—C5	−178.39 (9)	C1—N1—C12—C11	179.80 (10)
C2—C1—C6—C5	4.01 (16)	C1—N1—C12—C7	0.13 (11)
C4—C5—C6—C1	10.21 (14)	C10—C11—C12—N1	−179.93 (10)
C4—C5—C6—C7	−168.79 (10)	C10—C11—C12—C7	−0.29 (15)
C1—C6—C7—C8	−179.49 (10)	C8—C7—C12—N1	179.32 (9)
C5—C6—C7—C8	−0.36 (18)	C6—C7—C12—N1	0.36 (11)
C1—C6—C7—C12	−0.71 (11)	C8—C7—C12—C11	−0.39 (15)
C5—C6—C7—C12	178.41 (9)	C6—C7—C12—C11	−179.36 (9)

### 6-Methoxy-1,2,3,4-tetrahydrocarbazole (II) Hydrogen-bond geometry (Å, º)

*Cg*1 and *Cg*2 are the centroids of the C7–C12 and N1/C1/C6/C7/C12 rings, respectively.

**Table:**

*D*—H···*A*	*D*—H	H···*A*	*D*···*A*	*D*—H···*A*
N1—H1···*Cg*1^i^	0.88 (1)	2.41 (1)	3.2645 (11)	150
C11—H11*A*···*Cg*2^i^	0.95	2.61	3.5018 (12)	146

Symmetry code: (i) −*x*+3/2, *y*+1/2, −*z*+3/2.
